# The genetics of atypical hemolytic uremic syndrome

**DOI:** 10.1007/s11825-018-0216-0

**Published:** 2018-12-21

**Authors:** Wouter J. C. Feitz, Nicole C. A. J. van de Kar, Dorothea Orth-Höller, Lambert P. J. W. van den Heuvel, Christoph Licht

**Affiliations:** 1 Department of Pediatric Nephrology, Amalia Children’s Hospital, Radboud Institute for Molecular Life Sciences, Radboudumc, Nijmegen, The Netherlands; 2 Cell Biology Program, Research Institute, The Hospital for Sick Children, Toronto, ON, Canada; 3 Division of Hygiene and Medical Microbiology, Medical University of Innsbruck, Innsbruck, Austria; 4 Department of Development and Regeneration, Department of Pediatric Nephrology, KU Leuven, Leuven, Belgium; 5 Division of Nephrology, The Hospital for Sick Children, Toronto, ON, Canada; 6 Department of Pediatrics, University of Toronto, Toronto, ON, Canada

**Keywords:** Alternative complement pathway, Complement system, Endothelium, Microangiopathic hemolytic anemia, Thrombotic microangiopathy, Alternativer Komplementweg, Komplementsystem, Endothel, Mikroangiopathische haemolytische Anaemie, Thrombotische Mikroangiopathie

## Abstract

Atypical hemolytic uremic syndrome (aHUS) is a disorder characterized by thrombocytopenia and microangiopathic hemolytic anemia due to endothelial injury. aHUS is felt to be caused by defective complement regulation due to underlying genetic mutations in complement regulators or activators, most often of the alternative pathway. Mutations causing aHUS can be subdivided into two groups, loss of function mutations (affecting factor H, factor H-related proteins, membrane co-factor protein, and factor I), and gain of function mutations (affecting factor B and C3). As more information becomes available on the relationship between specific mutations and clinical outcome, complete genetic workup of aHUS patients becomes more and more important.

In this review, we will discuss the genetic background of aHUS, the role of complement for aHUS pathogenesis, and the different groups of specific mutations known to be involved in the pathogenesis of aHUS.

## Zusammenfassung

Das atypische hämolytisch-urämische Syndrom (aHUS) ist durch Thrombozytopenie und mikroangiopathische hämolytische Anämie im Kontext einer Endothelzellschädigung charakterisiert. Die Ursache des aHUS ist ein Defekt in der Regulation des Komplementsystems, der durch genetische Mutationen von Komplementregulatoren oder -aktivatoren meist im Bereich des alternativen Wegs verursacht wird. Krankheitsverursachende Mutationen können in zwei Gruppen eingeteilt werden: „loss of function“ (Komplementregulatoren Factor H, Factor H related proteins, Membrane-cofactor-protein und Factor I) und „gain of function“ Mutationen (Komplementaktivatoren Factor B und C3). Die enge Genotyp-Phänotyp-Korrelation unterstreicht die Notwendigkeit einer vollständigen genetischen Diagnostik.

Im vorliegenden Review werden die Bedeutung des Komplementsystems und der spezifischen genetischen Ursachen, die für die Pathogenese des aHUS relevant sind, diskutiert.

## Introduction

### Thrombotic microangiopathy

Thrombotic microangiopathy (TMA) defines a spectrum of disorders characterized by thrombocytopenia and microangiopathic hemolytic anemia (MAHA) due to endothelial injury. TMA was first described by Moschcowitz in 1924 [1–3] and is an ubiquitous disease that can involve multiple organs including the brain, heart, lungs, gastrointestinal tract, and kidneys [1]. In the two most common types of TMA, the major target organs are the central nervous system (CNS) in thrombotic thrombocytopenic purpura (TTP), and the kidneys in hemolytic uremic syndrome (HUS) [1]. In the majority of cases TTP is caused by IgG and IgM autoantibodies impairing the function of ADAMTS13, a disintegrin and metalloprotease with a thrombospondin type 1 motif, member 13 (i. e., Morbus Moschcowitz). In a small group of patients, TTP is caused by mutations in the gene encoding for ADAMTS13 (i. e., Upshaw–Schulman syndrome) [1, 3–6]. Both the acquired and genetic forms of TTP cause impaired ADAMST13 function and lead to a severely decreased (i. e., <10%) ADAMTS13 activity. Physiologically, ADAMTS13 specifically cleaves the ultra-large von Willebrand factor (VWF) multimers released from the vascular endothelium into smaller multimers, and ADAMTS13 deficiency results in the presence of ultra-large vWF in the circulation, leading to platelet adhesion and thrombus formation [1, 3, 4].

By contrast, HUS has various etiological factors. Most cases of HUS are caused by an infection with Shiga toxin (Stx)-producing *Escherichia coli* (STEC-HUS or eHUS) [1, 7–9]. After a prodromal phase of often bloody diarrhea, the Stx can enter the bloodstream and lead to damage of the vascular endothelium with subsequent thrombus formation and renal failure. Although most HUS cases occur in children and are the consequence of an infection with Stx-producing *Escherichia coli *(STEC), HUS can also be primarily caused by a dysregulation of the complement system (atypical HUS [aHUS]) or occur secondary to underlying conditions such as infections, drugs, pregnancy, and metabolic diseases (secondary TMA) [8, 10, 11].

In aHUS, genetic susceptibility and triggering events such as respiratory tract and gastrointestinal infections combine in the manifestation of a TMA event (“multiple-hit hypothesis”) [5, 8]. The fact that a first aHUS event can occur only later in life indicates the need for an additional trigger. aHUS is rare, with an incidence of approximately 0.5 million per year [12], can affect all age groups, runs in families, and comes with a significantly more severe phenotype than STEC-HUS. It is caused by defective complement regulation, specifically by mutations or autoantibodies affecting complement regulators or activators [5, 8, 10, 11, 13, 14], most often of the alternative pathway [2].

Endothelial injury in aHUS patients ranges from only mild endothelial swelling to full occlusion of the vessel due to platelet adhesion and thrombus formation. Renal biopsies from aHUS cases show the classical pattern of TMA: glomerular capillary wall thickening, mesangiolysis, platelet/fibrin deposition, and narrowing of the vessels; on blood film typically (but not necessarily) erythrocyte fragments are seen [4, 11]. Chronic TMA lesions, such as double contours of peripheral capillary walls, new subendothelial basement membrane, and subendothelial flocculent material, may be seen by electron microscopy [10].

The focus of this review will be on aHUS, the role of complement and the involvement of genetics in the pathogenesis of this rare disease.

### The complement system

**Fig. 1:**
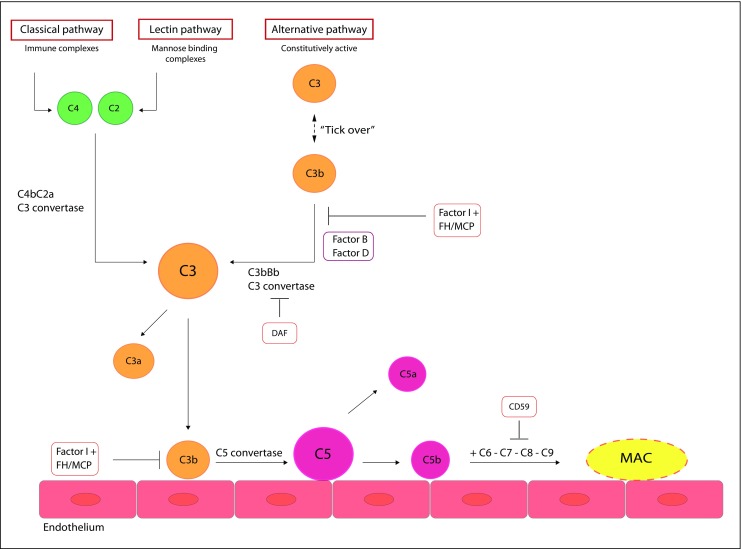
The complement system. The complement system can be activated by three pathways: the classical, lectin or alternative pathway. All three activation pathways merge in the activation of complement C3. The terminal complement pathway is activated via the activation of complement C5, resulting in the formation of the membrane attack complex. **FH** factor H, **MCP/CD46** membrane cofactor protein, **DAF/CD55** decay accelerating factor, **MAC/C5b‑9** membrane attack complex

The complement system (Fig. 1) is part of innate immunity. Its main functions are immune complex clearing, chemotaxis for recruiting inflammatory cells, opsonization of invading microbes, phagocytosis of foreign particles, and cell lysis [11, 15]. The complement system consists of >30 proteins [4] and can be activated by three pathways: the classical (CP), lectin (LP), and alternative pathway (AP). Although the classical and lectin pathways are inducible via immune complexes (classical) or the presence of certain carbohydrate sequences, for example, those found on bacterial surfaces (mannose-binding lectin [MBL] or ficolin to carbohydrate sequences), the alternative pathway is constitutively active and therefore requires tight regulation [5, 8, 15–17]. All three activation pathways merge in the activation of complement C3 via the C3 convertase complex C3bBb (for the AP) or C4b2a (for the CP and LP) resulting in the formation of the anaphylatoxin C3a and the generation of the opsonin C3b. Via formation of C5 convertase (AP: C3bBbC3b or CP: C4bC2aC3b, respectively), C3b can induce the terminal complement pathway, resulting in the formation of the anaphylatoxin C5a and the membrane attack complex (MAC, C5b-9), causing cell lysis [5, 8, 15–17].

**Fig. 2:**
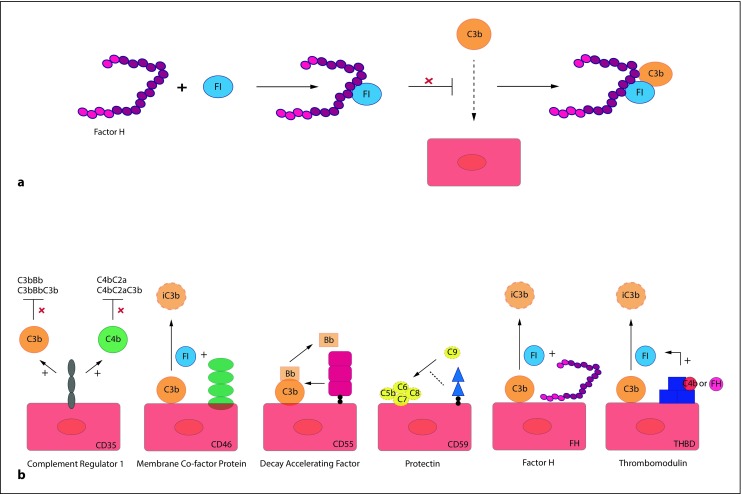
Fluid phase and membrane-bound regulators of the complement system. **a** FH is the most important fluid phase regulator of the complement system. FH acts as a cofactor for FI-mediated C3 cleavage. FH also prevents C3b from binding to the endothelial cell surface. In addition, FH facilitates the decay of the AP C3 convertase. **b** Membrane bound regulators protect cells from (over)activation of complement on the cell surface. Complement regulator 1 (CD35) binds C3b and/or C4b and prevents the formation of C3 and C5 convertases, membrane co-factor protein (MCP, CD46) inactivates C3b and C4b by acting as a cofactor, decay-accelerating factor (DAF, CD55) accelerates the decay of the AP C3 convertase C3bBb, protectin (CD59) blocks the formation of MAC/C5b-9, and thrombomodulin (THBD, CD141) works as a cofactor for FH and C4b, resulting in the inactivation of C3b. **FI** factor I, **FH** factor H, **THBD/CD141** thrombomodulin

**Fig. 3:**
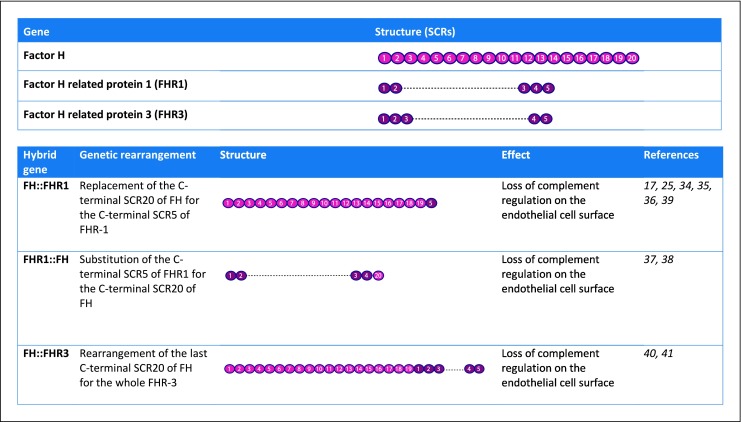
FH::FHR hybrid genes related to aHUS. FH::FHR1, FHR1::FH, and FH::FHR3 hybrid genes are known to be related to aHUS and cause loss of complement regulation on the endothelial cell surface. Pink SCRs are SCRs from FH, whereas dark purple SCRs are related to FHR1 or FHR3. **FH** factor H, **FHR** factor H-related protein, **SCR** short consensus repeat

Serum and membrane-anchored complement proteins combine to limit complement activation both temporally and spatially with factor H (FH) being the most important fluid phase regulator. FH belongs to the factor H family of proteins and consists of 20 short consensus repeats (SCRs). The N‑terminus of FH with SCRs1–4 harbors the cofactor activity for factor I (FI)-mediated C3b cleavage (cofactor activity) and accelerates the natural decay of C3 convertases (decay accelerating activity). The C‑terminus of FH with SCRs19–20 mediates the binding to C3b/C3d, heparin, glycosaminoglycans, and to the cell surface (Fig. 2a; [5, 6, 11]). Structurally and possibly functionally similar to FH are the proteins of the group of factor H-related proteins (FHRs), including FHR1, FHR2, FHR3, FHR4, and FHR5 [18]. The two C‑terminal SCR domains of FHR1–5 are (almost) identical to the FH C‑terminus [18]. FHR1 lacks the regulatory function of FH; however, it has been shown to control the complement cascade at a later step by blocking C5 convertase activity and the formation of MAC/C5b-9 [18]. Since the coding region of the FHR genes is located next to the FH gene (i. e., chromosome 1q32) and carries a high rate of sequence homology, it is highly susceptible for genetic rearrangements between FH and FHRs, leading to the formation of FH::FHR hybrid genes [7, 11, 14, 18, 19]. The most relevant hybrid genes for aHUS are composed of FH and FHR1 or FHR3 (Fig. 3; [5, 18]) and can lead to products with decreased local complement regulation on the endothelial cell surface [10, 14, 20].

Membrane-anchored proteins on the cell surface, including complement receptor 1 (CR1, CD35), membrane cofactor protein (MCP/CD46), decay-accelerating factor (DAF/CD55), and protectin (CD59) protect cells from the over-activation of complement on their surfaces [5, 9, 16]. Membrane-anchored proteins inactivate bound C3b and C4b by acting as cofactor (MCP/CD46), accelerate the natural decay of C3 and C5 convertases (DAF, CD55), or by blocking the formation of MAC/C5b-9 (protectin/CD59; Fig. 2b; [9, 10]).

The amount of activated complement is dynamically regulated, depends on multiple factors and varies per organ. The microenvironment of the kidneys with high blood flow, high concentrations of complement proteins, endothelial fenestrae with uncovered glomerular basement membrane and the local production of complement proteins likely results in a higher susceptibility of the kidney for complement-mediated insults.

### Genetics

**Fig. 4:**
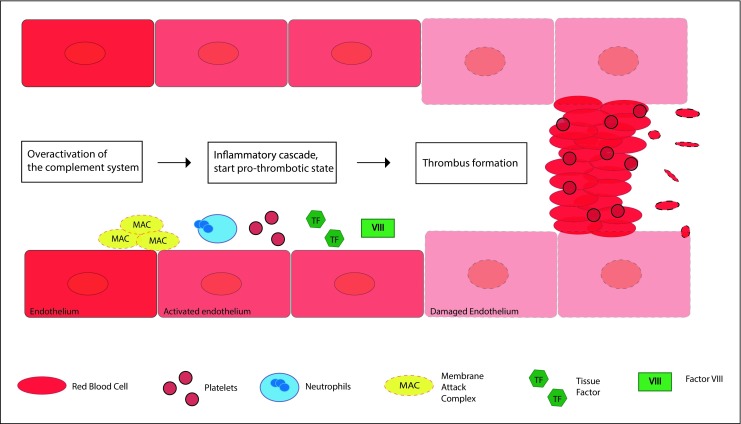
Schematic overview of the underlying pathophysiology of aHUS. Endothelial activation and damage in the glomerulus of the kidney is the central pathologic feature in aHUS. Over activation of the complement cascade results in the formation of MAC/C5b-9 on the endothelial cell surface. Next to endothelial activation, endothelial damage stimulates the inflammatory cascade with involvement of neutrophils and the start of the coagulation cascade with platelets, tissue factor and factor VIII. This results in thrombus formation. **aHUS** atypical hemolytic uremic syndrome, **MAC/C5b-9** membrane attack complex, **TF** tissue factor, **VIII** factor VIII

The main pathological feature of aHUS is endothelial injury. Over activation of the complement system caused by underlying genetic mutations, results in an increased level of MAC/C5b-9 deposition on the endothelial cell surface causing endothelial cell activation and damage [4, 7–9, 11]. Endothelial activation and damage results in inflammation and activation of the coagulation cascade with thrombus formation as result (Fig. 4; [8]). As genetic mutations predispose for complement dysregulation, screening of the genetic complement profile is important in aHUS patients.

The general tool widely used to detect genetic variants is sequence analysis of the coding regions of candidate genes in aHUS patients. This allows for a comprehensive analysis of all genetic variations in a candidate gene or set of candidate genes. With the development of next-generation sequencing (NGS) technologies, tens to hundreds of genes can be effectively analyzed simultaneously. However, whereas candidate gene sequencing is a targeted approach, whole exome sequencing (WES) or whole genome sequencing (WGS) can interrogate sequence variants in all coding regions/exons (WES) or even the entire genome (WGS). The sequence homology of some complement genes (for example, the genes from the *FH* operon) results in ambiguous read alignments from NGS data sets. For this reason, a diagnostic routing frequently consists of a combination of amplicon-based sequencing of the *FH* operon (at least SCR20 and the complete coding region of *FHR1-5*) and deep intronic single nucleotide polymorphisms (SNPs) of aHUS-associated haplotypes, in combination with an NGS-based gene panel aiming to establish sequence variants in aHUS patients. In the near future this will probably be replaced by NGS once the technology has become more cost effective.

In the human genome next to the *FH* gene, genes encoding FHRs1–5 are located in tandem next to FH on chromosome 1q31.3. All these genes originate from *FH* through gene duplication events [21]. The *FH-FHRs* loci contain several segmental duplications, making them prone to genetic structural rearrangements due to allelic and non-allelic homologous recombination events. This has led to copy number polymorphisms, for example, the common deletion that results in the loss of *FHR1–FHR3 (Rs 6677604*). In aHUS patients, genetic structural rearrangement in the *FH* operon may be observed, resulting in different *FHR *hybrid genes. For genetic diagnostics, this copy number variation in the *FH-FHRs* genomic region can be evaluated using multiplex ligation-dependent probe amplification (MLPA) [22]. In the future, novel long-read NGS technology may replace (part of) this MLPA approach.

#### Interpretation of genetic results

Functional genomics will become more and more important in unraveling the functional consequences of genetic aberrations. The likely functional impact of identified sequence variations can be predicted using *in silico* software (such as PolyPhen, Align GVD, and SIFT), but these are not always accurate. Several established mutations identified in aHUS patients have dbSNP numbers (numbers of the Short Genetic Variations database), as they have been identified in large NGS projects (for example, the p.Arg1210Cys and rs12191309). For most functional analysis methods that are available today (such as crystallography, Biacore, hemolytic assays) and/or cofactor assays, recombinant protein production and purification or isolation of mutant proteins from patients’ blood is needed, which may be a time-consuming and challenging matter. Because of its complexity, it should be performed in centers of expertise.

#### Specific genetic mutations in aHUS

**Fig. 5:**
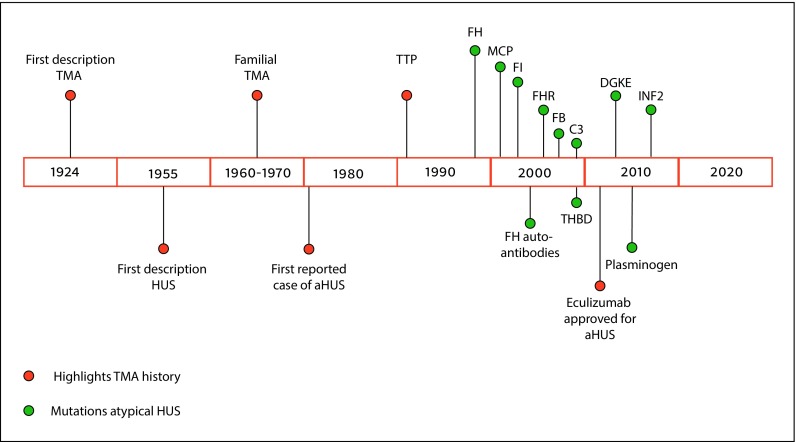
Historical timeline of the main highlights of TMA and aHUS. Timeline of major TMA- and aHUS-related findings starting in 1924 with the first description of TMA by Moschcowitz [1–3]. Orange dots are related to the main events in the story of TMA, whereas green dots are related to mutations found in aHUS. **TMA** thrombotic microangiopathy, **HUS** hemolytic uremic syndrome, **aHUS** atypical hemolytic uremic syndrome, **TTP** thrombotic thrombocytopenic purpura, **FH** factor H, **MCP/CD46** membrane cofactor protein, **FI** factor I, **FHRs** factor H-related proteins, **FB** factor B, **THBD/CD141** thrombomodulin, **DGKE** diacylglycerol kinase epsilon, **INF2** inverted formin-2

The first report on a patient with aHUS was published in 1981 [23], when Thompson and Winterborn described an 8‑month-old Asian boy presenting with symptoms of HUS and very low C3 levels but normal C4 levels [23]. This case was published before the era of genetic screening, and it was only later that Warwicker et al. (1998) linked a case of familial aHUS with a FH mutation [24]. The discovery of mutations of several other genes, encoding for complement-activating or -regulating proteins, followed (Fig. 5).

Mutations causing aHUS can be subdivided into *loss of function* mutations of complement regulators (FH, FHRs, MCP/CD46, FI), or *gain of function* mutations of complement activators (FB, C3). In addition, FH autoantibodies can be identified, which in most patients coincide with the presence of a homozygous deletion in FHR1 and/or FHR3 [2, 6–8, 10, 11, 14–16, 25–27].

#### Loss of function mutations

*Loss of function mutations* are mutations that result in the loss of function of fluid phase or membrane-anchored complement regulators and cause ongoing excessive complement activation with subsequent endothelial cell injury. Mutations involved affect the genes encoding for FH, FHRs, MCP/CD46, and FI [8, 14]. Although these mutations lead to uncontrolled activation of complement proteins on surfaces such as the vascular endothelium, complement regulation in the fluid phase remains intact in most cases. The variation in severity of complement regulation may be one explanation for the normal systemic C3 levels seen in many of aHUS patients. Thus, normal plasma C3 levels can be found in aHUS patients, and do not rule out the presence of genetic abnormalities.

The most frequent mutations seen in aHUS are heterozygous and affect FH, which is involved in 21–25% of cases [5, 11, 12, 14, 28, 29]. Most of the mutations in FH are found in SCRs 19 and 20 [5, 14, 25]. In recently published data from the Global aHUS Registry, 100 out of 482 (21%) patients carried an FH mutation, 37 out of 395 (9%) an MCP/CD46 mutation, and 26 out of 406 (6%) an FI mutation [12]. Krishnappa et al. (2018) published a meta-analysis of 259 aHUS cases published between 2005 and 2015. This analysis showed an even higher rate of 50% of FH mutations in aHUS patients (69 out of 139) [30]. In addition, the analysis showed that 35% of patients had FHR1 mutations, 22.8% had MCP/CD46 mutations, and 16.6% had FI mutations [30]. Osborne et al. (2018) analyzed over 3,500 patients with aHUS and C3 glomerulopathy and reported 371 genetic variants in patients diagnosed with aHUS [19].

Fremeaux-Bacchi et al. [13] analyzed 214 patients with aHUS in a French nationwide cohort between 2000 and 2008. They reported a total of 60.2% mutations with 22.5% FH mutations (45 of 200 cases). Mutations in FHR1, MCP/CD46 and FI genes occurred at frequencies of 4.5%, 10%, and 9% respectively [13]. An American cohort of 144 patients screened by Maga et al. (2010) found that 27% had an FH mutation, 5% had an MCP/CD46 mutation, and 8% had an FI mutation [29], whereas Geerdink et al. (2012) screened a Dutch and Belgium cohort consisting of 45 pediatric patients diagnosed with aHUS and found 5 mutations in FH (11%), 4 mutations in MCP/CD46 (9%), and 3 mutations in FI (7%) [31].

Studies have shown that some aHUS patients carry one or more of the defined risk haplotypes (mostly SNPs) of the genes encoding for FH, FHR1 or MCP/CD46, in addition to a complement mutation [4, 13, 17, 32, 33]. In combination with a genetic mutation, these risk haplotypes lead to an increased risk for the development of aHUS, but a haplotype itself is seen as a susceptibility factor rather than the direct cause of the disease [13, 17, 32, 33].

As mentioned above, aHUS can also be due to FH::FHR hybrid genes, which are caused by rearrangements between FH and FHR genes. There are only a few such cases described in the literature, and the incidence of hybrid genes in aHUS is estimated to be around 1–5% [7, 10]. The first case was published in 2006 by Venables et al. [34]. They described a case of a family with multiple members with aHUS, and used cDNA analysis for genetic screening. This family screened positive for an FH::FHR1 hybrid gene [34]. In 2010, Noris et al. published a cohort of 273 patients with aHUS, 3 of whom (1%) had FH::FHR1 hybrid genes [25]. Maga et al. (2011) reported a patient with aHUS after 11 months of age. Genetic screening revealed a mutation in MCP/CD46, and MLPA assays showed a FH::FHR1 hybrid gene [35]. Bresin et al. (2013) screened 795 aHUS patients and found 1 patient with a FH::FHR1 mutation [17], whereas in the same year, Le Quintrec et al. (2013) studied 57 aHUS patients retrospectively after renal transplantation and described 6 patients with a FH::FHR1 hybrid gene in their cohort [36]. Eyler et al. (2013) described a case report of a 14-year-old girl with aHUS caused by a FH::FHR1 hybrid gene [37], whereas Valoti et al. [38] screened 154 aHUS patients using MLPA. They found 7 patients with a FH::FHR1 hybrid gene (4.5%) [38]. Goicoechea de Jorge et al. [39] did a retrospective genetic analysis of their aHUS cohort consisting of 513 patients, using MLPA. They found 9 patients with a FH::FHR1 hybrid gene [39].

FH::FHR3 hybrid genes are even less frequently found in aHUS patients than FH::FHR1 hybrid genes. Francis et al. (2012) screened a family with multiple cases of aHUS and found a FH::FHR3 hybrid gene [40], and Challis et al. (2016) described an 8‑month-old boy with aHUS in whom a FH::FHR3 hybrid gene was detected via MLPA [41].

#### Gain of function mutations

*Gain of function mutations* are mutations in the complement proteins FB and C3. Gain of function mutations lead to enhanced stability and thus activity of the alternative C3 convertase, which is resistant to regulation, resulting in the overproduction of the terminal complement pathway product MAC/C5b-9 [14].

Analysis of the Global aHUS Registry (2018) showed that 4 out of 275 patients (2%) had a mutation in FB, and 21 out of 331 (6%) had a mutation in C3 [12]. Fremeaux-Bacchi et al. (2013) found that 1.9% of 214 patients had FB mutations, and 8.4% of had C3 mutations in the French Nationwide cohort [13]. Maga et al. (2010) found 6 patients (4%) with FB mutations and 3 patients (2%) with C3 mutations in the American aHUS cohort consisting of 144 patients [29]. Geerdink et al. (2012) found that 2 related patients had an FB mutation (4%) and 4 patients had a C3 mutation (9%) in their cohort of 45 pediatric patients with aHUS [31].

Percentages of the different registries and cohorts are matching and indicate that gain of function mutations are less common than loss of function mutations in aHUS patients.

#### Thrombomodulin

Thrombomodulin (THBD/CD141) is a glycoprotein on the endothelial cell surface that has anti-coagulant properties and plays a role in the regulation of complement activation [6, 25]. Patients with THBD/CD141 mutations, degrade less C3b and generate less thrombin-activatable fibrinolysis inhibitor (TAFIa). TAFI is a carboxypeptidase responsible for the cleavage of the anaphylatoxins C3a and C5a [6, 26]. Mutations in *THBD/CD141* are found in 2–5% of patients with aHUS [6, 7, 12, 25, 26, 29]. Delvaeye et al. (2009) screened 152 patients with aHUS for mutations in *THBD/CD141* and found 6 different mutations in 7 patients (4.6%) [26]. Noris et al. (2010) screened 273 patients with aHUS and found 13 carriers of a single heterozygous *THBD/CD141* mutation (4.7%) [25]. Furthermore, they found that patients with *THBD/CD141* mutations, along with mutations in FH, had the earliest onset and highest mortality rate of patients with aHUS. They also showed the involvement of THBD/CD141 in complement regulation by demonstrating its cofactor activity to FH and the decreased capacity of mutated THBD/CD141 to inactivate C3b [26]. Maga et al. (2010) screened 144 patients and found 4 patients (3%) with an *THBD/CD141 *mutation in the American aHUS cohort [29]. Fremeaux-Bacchi et al. (2013) found no *THBD/CD141* mutations in the French Nationwide cohort [13], whereas the Global aHUS Registry (2018) showed 4 patients with mutations in *THBD/CD141* out of the 193 patients tested (2%) [4].

#### Noncomplement-related genetic mutations in aHUS

Because of the extended use of genetic testing to analyze large patient cohorts, on rare occasions new genetic mutations are found. In a small group of patients with the clinical diagnosis aHUS, a rare mutation is found in non-complement-related proteins. Examples are diacylglycerol kinase epsilon (DGKE), inverted formin-2 (INF2), and plasminogen (PLG).

DGKE is a protein involved in cell metabolism, and is responsible for the catalyzation of diacylglycerol to phosphatidic acid, both lipids playing an important role in cell lipid signaling. DGKE is also responsible for the modulation of protein kinase C (PKC) activity [8, 14]. DGKE mutations result in the activation of PKC, which stimulates pro-thrombotic factors such as VWF and tissue factor [3, 42]. Patients with homozygous DGKE mutations typically present with a TMA episode during the first year of life, develop proteinuria, and progress to end stage renal disease during their second to third decade of life [3, 8, 9, 42]. DGKE was first described by Lemaire et al. in 2013, who found mutations in 9 patients using exome sequencing [42]. Mutations in DGKE are rare, only a few cases have been published [4], and a link to complement has not been identified [2–4, 8, 9, 43].

INF‑2 belongs to a family of proteins called formins. It plays an important role in the regulation of certain cytoskeletal functions as it accelerates actin polymerization and depolymerization [44]. Challis et al. [41] used WES to analyze 28 families with cases of aHUS and used Sanger sequencing to analyze 161 sporadic aHUS cases. In 2 families, they identified mutations in the gene encoding for INF2 [44], whereas no INF2 mutations were found in the sporadic aHUS cases. Functional analysis of the mutations showed modified INF2 localization and disrupted cytoskeleton [44]. Members of one of the families carried a homozygous FH haplotype and was homozygous for a MCP/CD46 risk haplotype, whereas members of the other family carried a homozygous MCP/CD46 risk haplotype and one copy of a FH haplotype [44].

PLG is another protein found to be related to aHUS, but not related to the complement cascade. PLG is encoded by the PLG gene [22] and becomes converted into plasmin during clot formation. Plasmin is important for the resolution of blood clots and thus the regulation of the coagulation pathway. Decreased production of PLG results in decreased plasmin levels with diminished dissolution of blood clots and dysregulation of the coagulation pathway, eventually resulting in thrombus formation. Bu et al. [22] used targeted genomic enrichment and massive parallel sequencing to screen 36 patients with aHUS. They found 4 patients with a variant in the PLG gene [22] and concluded that those variants were related to the disease course of aHUS.

#### Clinical utility of genetic information

Published data have shown and described that the different genetic mutations known to be involved in aHUS come with a different age at disease onset, phenotype/genotype relationship, and risk of recurrence (Table 1; [5, 7, 10–14, 25, 28, 30, 31, 33, 36, 43, 45]). Functional studies of disease-specific genetic variants will help to identify pathogenic mechanisms and guide development of focused therapies. Knowledge of the pathological implications of complement genetic makeup will allow for an individualized assessment of disease predisposition, facilitating the implementation of personalized and preventive medical procedures and personalized treatment plans.

**Table 1:** Complement-related genetic mutations in aHUS. Various clinical outcomes and the relationship with the different complement-related genetic mutations involved in aHUS

		Function in complement system	Frequency in aHUS (%)	ESRD after 5 years (%)	Recurrence (%)	Recurrence after kidney transplantation (%)	References
FH	Factor H	Co-factor for factor I	21–25	70–80	30–50	68–90	[5, 8, 10–14, 19, 25, 28–31]
MCP/CD46	Membrane cofactor protein	Membrane-bound complement regulator	5–22.8	10–50	58–90	11–20	[5, 8, 10–13, 29–31]
FI	Factor I	Inactivation of C3b and C4b	6–16.6	45–60	10–30	70–80	[5, 8, 10, 12, 13, 29–31]
FB	Factor B	Allows the formation of C3 and C5 convertases	1.9–4	70	Rare	Rare	[5, 8, 10, 13]
C3	Complement C3	Necessary for complement cascade activation	6–9	45–65	50	40–50	[5, 8, 10, 12, 13]
FHRs	Factor H‑related proteins	Circulating proteins similar to factor H associated with autoantibodies against FH	4.5–35	30–63	23–60	20	[8, 10, 12, 13, 30]
FHR hybrid genes	Factor H, Factor H-related proteins	See function FH and FHRs	1–5	–	–	–	[8, 10]
THBD/CD141	Thrombomodulin	Degradation C3b	2–5	53–60	23–30	Rare	[5, 8, 10, 13]

**FH** factor H, **MCP/CD46** membrane cofactor protein, **FI** factor I, **FB** factor B, **FHR** factor H-related proteins, **THBD/CD141** thrombomodulin, **aHUS** atypical hemolytic uremic syndrome, **ESRD** end-stage renal disease

## Conclusion

In this review, we have described the currently known mutations involved in aHUS pathogenesis. As more information becomes available about the relationship between specific mutations and the course of disease, complete genetic workup becomes increasingly important in patients with aHUS. Eventually, the underlying genetic mutation will play an important role in the choice of personalized treatment options.

## Abbreviations

aHUS: Atypical hemolytic uremic syndrome
AP: Alternative pathway (complement system)
CNS: Central nervous system
CP: Classical pathway (complement system)
CR1: Complement receptor 1
DAF: Decay accelerating factor
DGKE: Diacylglycerol kinase epsilon
eHUS: *E. coli* hemolytic uremic syndrome
FB: Factor B
FH: Factor H
FHR: Factor H-related protein
FI: Factor I
HUS: Hemolytic uremic syndrome
INF2: Inverted formin-2
LP: Lectin pathway (complement system)
MAC: Membrane attack complex
MAHA: Microangiopathic hemolytic anemia
MBL: Mannose binding lectin
MCP: Membrane cofactor protein
MLPA: Multiple ligation-dependent probe amplification
NGS: Next-generation sequencing
PKC: Protein kinase C
SCR: Short consensus repeat
SNP: Single nucleotide polymorphisms
STEC: Shiga toxin-producing *E. coli*
STEC-HUS: Shiga toxin-producing *E. coli* hemolytic uremic syndrome
Stx: Shiga toxin
TAFIa: Thrombin activatable fibrinolysis inhibitor a
THBD: Thrombomodulin
TMA: Thrombotic microangiopathy
TTP: Thrombocytopenic purpura
VWF: Von Willebrand factor
WES: Whole exome sequencing
WGS: Whole genome sequencing
